# A Lightweight Deep Learning Approach for Real-Time Detection of Egg-Laying Behavior and Hatching Eggs in Breeding Ducks

**DOI:** 10.3390/ani16213334

**Published:** 2026-10-22

**Authors:** Shida Zhao, Xiaoyue Hu, Lihua Li, Zixuan Zhou, Hao Xue, Xiaoliu Zheng, Xingmao Yuan, Yan Chen, Ruodong Zhi

**Affiliations:** 1 Institute of Agricultural Information and Economy, Hebei Academy of Agriculture and Forestry Sciences, Shijiazhuang 050051, China; 2 College of Mechanical and Electrical Engineering, Hebei Agricultural University, Baoding 071001, China; 3 Hebei Agriculture Mechanization Institute Co., Ltd., Shijiazhuang 050051, China; 4 School of Mechanical Engineering, Wuhan Polytechnic University, Wuhan 430023, China

**Keywords:** breeding duck, behavior recognition, hatching egg detection, YOLO, reproductive performance

## Abstract

Egg production rate and laying behavior are important indicators of reproductive performance in breeding ducks. Manual monitoring is time-consuming and labor-intensive, and litter, small eggs, variable postures, and occlusion complicate detection. This study proposed DPG-YOLO to detect egg-laying behavior and hatching eggs. Based on YOLO26, the DE-C3K2 module was reconstructed to enhance fine-grained feature extraction. PPA was incorporated to strengthen multi-scale semantic modeling, and C3Ghost was adopted to optimize neck feature fusion and reduce redundant computation. DPG-YOLO achieved precision, recall, and mAP50 values of 96.8%, 96.3%, and 97.4%, respectively, exceeding YOLO26 by 4.6, 4.8, and 5.0 percentage points. The model required 7.6 GFLOPs, occupied 7.1 MB, and processed 124.1 frames/s. Automatically estimated egg production rate and laying behavior frequency were significantly and positively correlated with manually calculated values (*r_s_* = 0.987 and 0.964, *p* < 0.05). Neither indicator differed significantly between the two methods (*p* > 0.05). Thus, DPG-YOLO combines detection accuracy, real-time performance, and statistical reliability to support automated monitoring of reproductive performance, precision feeding management, and digitalization of breeding duck production.

## Simple Summary

Egg production rate and laying behavior help farmers assess reproductive performance of breeding ducks. These indicators are usually recorded by hand, which requires considerable labor and is unreliable in complex housing conditions. We developed a lightweight computer model based on deep learning to detect laying behavior and eggs intended for hatching in overhead videos of breeding ducks. The model was adapted from an existing image detector to capture small details and objects of different sizes while limiting unnecessary computation. It was trained and tested using 19,630 images from a commercial breeding duck farm. It achieved a mean average precision of 97.4%, a measure of detection performance, and processed 124.1 frames per second. Its estimates of egg production rate and laying frequency agreed closely with manual records. This approach enables continuous, non-invasive monitoring of reproductive status and supports precise flock management and the use of digital technologies in breeding duck production.

## 1. Introduction

China is a major waterfowl producer, and breeding duck production underpins its duck industry. The reproductive performance of breeding ducks directly determines the supply of commercial ducklings and the industry’s economic returns. Egg production rate and laying behavior frequency are key indicators of reproductive performance and production status, which inform duck breeding and precision feeding management [1,2]. These indicators are currently obtained mainly through manual inspection, egg counting, and behavioral observation. These approaches require considerable labor, are highly subjective and poorly reproducible, and limit continuous monitoring. Complex litter backgrounds, variable illumination, and occlusion among ducks further reduce the efficiency and reliability of manual recordings. Therefore, technologies that automatically and accurately record egg-laying behavior and continuously count hatching eggs are important for evaluating reproductive performance, managing feeding precisely, and digitalizing duck breeding production [3].

Recent advances in machine vision and deep learning have created new approaches to these problems. Object detection has progressed from two-stage to one-stage approaches. Two-stage detectors, such as Faster R-CNN [4], achieve high accuracy; however, their slow inference makes them unsuitable for real-time monitoring. In contrast, the end-to-end design and efficiency of one-stage YOLO detectors have driven continued development, including YOLOv9 [5], YOLOv10 [6], YOLOv11 [7], and YOLOv12 [8]. Real-time detectors such as RT-DETR [9] have also demonstrated competitive performance. Successful poultry applications of YOLO include laying hen behavior recognition [10], free-range duck egg recognition and localization [11,12,13], and egg detection and counting in cage-rearing systems [14,15]. Other applications include online egg crack detection [16] and automated detection of hatching information from hatching eggs [17,18]. These applications demonstrate the feasibility of deep learning for poultry monitoring. However, most studies have addressed either laying hen behavior or egg detection. Simultaneous automated detection of egg-laying behavior and hatching eggs in breeding ducks has received little attention. Hatching eggs and duck beaks occupy few image pixels and have poorly defined fine features, making them difficult to distinguish from litter texture, stains, and shadows. Target scales also vary considerably with distance from the camera. On-farm deployment generally relies on edge devices and requires a balance between accuracy, a lightweight design, and real-time performance. Existing general-purpose detectors struggle to meet these requirements simultaneously.

This study proposes DPG-YOLO, a lightweight multi-scale model for detecting egg-laying behavior and hatching eggs in breeding ducks, using YOLO26 [19] as the baseline. Here, hatching eggs refer to fertile eggs laid by breeding ducks and collected for incubation. Because fertile and non-fertile eggs are visually indistinguishable, the model detects all eggs visible in the nest boxes. In this flock, the farm collected essentially every egg laid in the nest boxes as a hatching egg. Floor eggs laid outside the nest boxes were rare and were excluded from the counts because the cameras and annotations covered only the nest-box areas. First, C3K2 was reconstructed using detail-enhanced convolution (DE-Conv), yielding DE-C3K2. This strengthened the extraction of fine-grained features, including hatching egg edges, duck beak contours, and local textures. Second, a Parallelized Patch-Aware Attention (PPA) module was incorporated before spatial pyramid pooling to strengthen multi-scale semantic modeling. Third, C3Ghost structures replaced conventional feature fusion modules in the neck network, reducing redundant computation and jointly optimizing detection accuracy and inference efficiency. Building on these changes, we established a method for automatically quantifying egg production rate and laying behavior frequency from video detections. Agreement between automatically and manually calculated values was then evaluated using Spearman’s rank correlation analysis and the Wilcoxon signed-rank test. The primary contribution lies in integrating these modules for the detection task and establishing a complete pipeline that jointly quantifies egg production rate and laying behavior frequency. This pipeline supports automated monitoring of reproductive performance and digitalization of duck production.

## 2. Materials and Methods

### 2.1. Data Acquisition and Processing for Egg-Laying Behavior and Hatching Eggs

**Figure 1:**
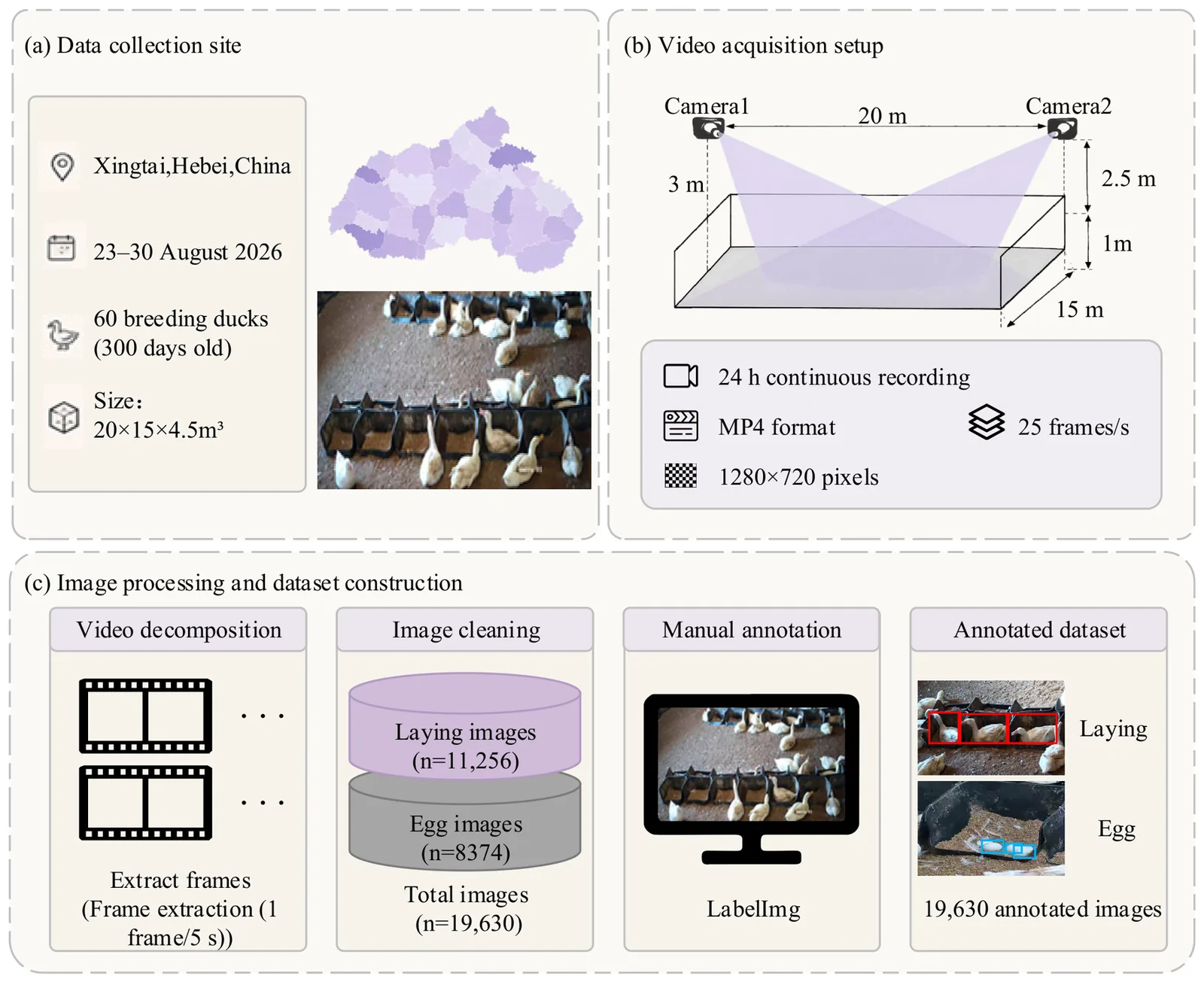
Data acquisition and processing pipeline: (**a**) experimental scene; (**b**) video acquisition setup; (**c**) data processing procedure.

Video and image data were collected in a dedicated experimental house at a commercial breeding duck farm in southern Xingtai, Hebei Province, China (38.830321° N, 115.446568° E). Data collection continued for 7 d, from 23 to 30 August 2026. The study used a floor-rearing system in a pen measuring 20 m long, 15 m wide, and 4.5 m high. The experimental flock comprised 60 female breeding ducks aged 300 days (Figure 1a). The house had a floor area of 300 m^2^, equivalent to 5 m^2^ per duck, and eight nest boxes arranged in two back-to-back rows of four. Routine feeding, drinking, and lighting regimes were maintained throughout data collection to capture egg-laying behavior under normal farm conditions.

Images were acquired using two Hikvision high-definition network cameras (DS-2CD3366WDV3-I, Hangzhou Hikvision Digital Technology Co., Ltd., Hangzhou, China). To capture the egg-laying process and hatching egg distribution as completely as possible, the cameras were mounted at different positions and viewing angles, approximately 3 m and 2.5 m above the laying area. Their complementary, partially overlapping fields of view covered the entire laying area without blind spots. Figure 1b illustrates the acquisition setup. Recordings continued for 24 h per day. Videos were stored in MP4 format at a resolution of 1280 × 720 pixels and a frame rate of 25 frames/s.

One frame was extracted from the videos every 5 s. Most raw frames from the 7-day recording period were discarded during image cleaning. Exclusion criteria included insects near the lens, blurring or ghosting caused by abrupt illumination changes and camera auto-exposure adjustments, severe overexposure or underexposure, and the absence of annotation targets. Frames without targets contained neither egg-laying behavior nor clearly visible hatching eggs. After cleaning, 11,256 egg-laying behavior images and 8374 hatching egg images remained, yielding a dataset of 19,630 annotated images.

Images were manually annotated using LabelImg [20] following an operational ethogram defined before annotation. At the image level, a breeding duck entering a nest box and remaining inside was labeled as egg-laying behavior. This label served as a visual proxy for oviposition rather than direct observation. At the event level, the temporal analysis described in Section 2.3.1 confirmed a valid egg-laying event only when an occupancy episode of valid duration was accompanied by a newly laid egg. A newly laid egg was defined as an egg detected in a nest region where no egg had been present before occupancy. Non-laying visits were retained as negative events with a validity value of 0 in Equation (4). Double occupancy was counted once per nest region. Successive visits and brief occlusions were handled using the event-merging threshold described in Section 2.3.1, and laying outside nest boxes was excluded by design. All annotations were manually cross-checked, and ambiguous cases were resolved in consultation with experienced breeding farm personnel. Figure 1c illustrates the annotation process.

### 2.2. Construction of the DPG-YOLO Model

**Figure 2:**
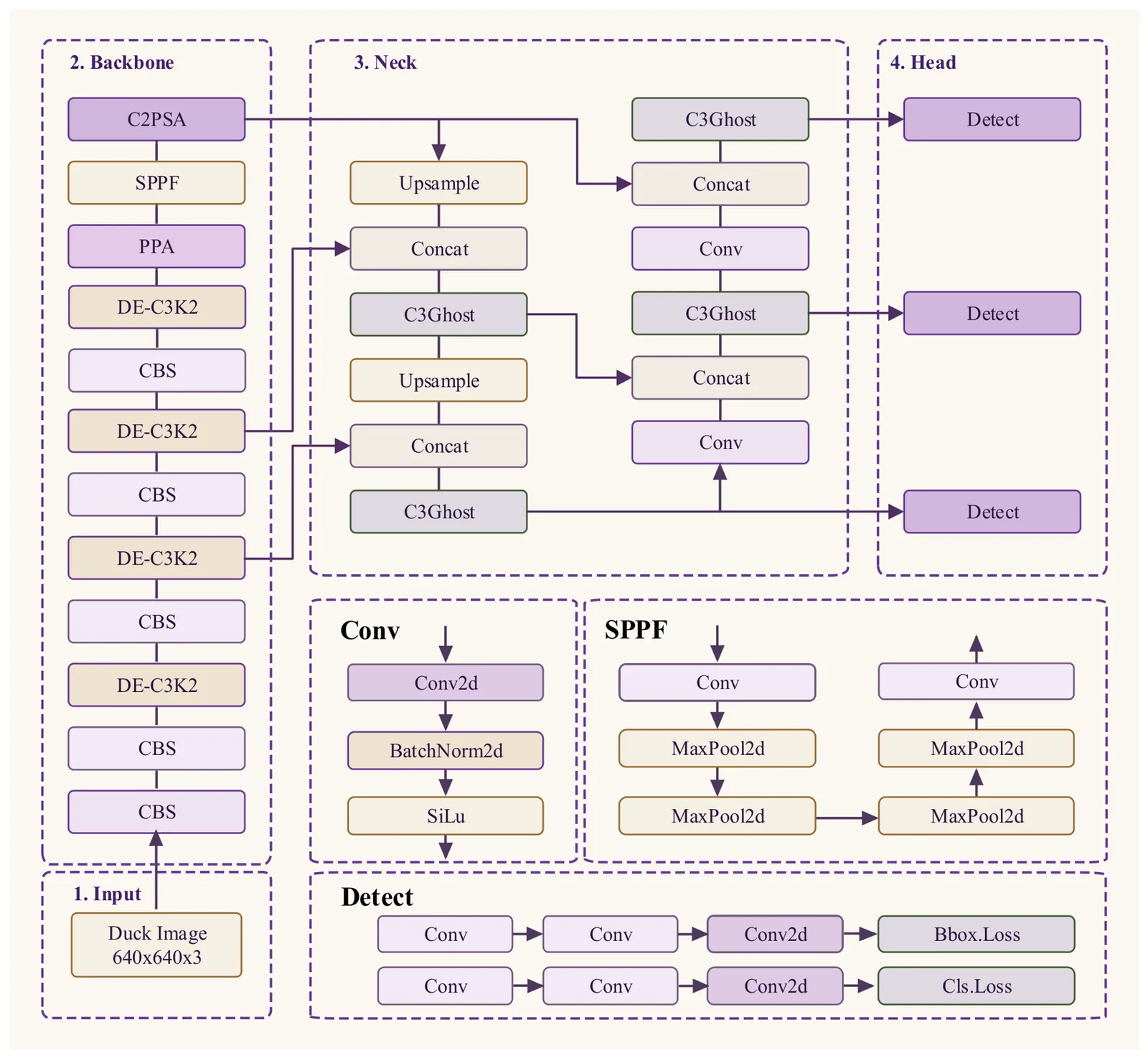
Overall network architecture of the proposed DPG-YOLO model.

DPG-YOLO was developed to address large differences in target scale, weak small-target features, and limited computing resources for deployment when detecting hatching eggs and egg-laying behavior in complex housing environments. It was designed as a lightweight multi-scale detection model based on YOLO26. All experiments used the official Ultralytics YOLO26 release (version 26.0.0, January 2026) with the n variant as the baseline architecture. In the backbone, DE-Conv enhanced detail extraction within conventional C3K2 convolutions, improving the capture of fine-grained information such as hatching egg edges, duck beak contours, and local textures. PPA was incorporated before the SPPF module to enhance the representation of duck behavior targets across scales. In the neck, C3Ghost replaced the four conventional C3K2 feature fusion modules to reduce parameter count and computational complexity. Figure 2 shows the overall architecture. Before network input, each 1280 × 720 frame was resized to 640 × 640 using aspect-ratio-preserving letterbox scaling with gray padding. This ensured consistent input dimensions without distorting target geometry. After downsampling, hatching eggs remained clearly distinguishable from the litter background.

#### 2.2.1. Optimization of Small-Target Feature Extraction

In duck behavior videos, hatching eggs, duck beaks, and regions where ducks interacted with feeders and drinkers were typically small. Duck beaks occupied a few pixels, and their appearance was readily affected by head posture, feather occlusion, motion blur, and illumination changes. Hatching eggs retained a relatively stable elliptical shape, but their edges could blend into backgrounds containing litter, eggshell stains, and shadows. During feature downsampling, conventional convolutions can capture high-level semantic information but have a limited capacity to retain small-target edges, local textures, and fine structures. The architectural enhancements described below were intended to capture these fine-grained cues, not to detect these elements as additional classes. DPG-YOLO outputs two classes: egg-laying behavior and hatching eggs.

We incorporated DE-Conv [21] into the YOLO26 backbone to enhance sensitivity to small-target details. Modifying the conventional convolution structure within C3K2 using DE-Conv yielded a module termed DE-C3K2.

DE-Conv combined standard convolutional feature extraction with difference feature enhancement, directing greater network attention to target edges, texture variations, and abrupt local grayscale changes. It retained a standard convolution branch and used differential convolutions with different directions or forms to extract local variations from feature maps. Equation (1) gives the resulting output.

(1) $$F_{\mathrm{de}}=F_{s}+\sum_{i=1}^{n}\alpha_{i}F_{d_{i}}$$

where *F_de_* denotes the output feature of DE-Conv; $F_{d_{i}}$ denotes the detail feature extracted by the *i*-th differential convolution branch; *α_i_* denotes the weighting coefficient of the corresponding branch; *n* denotes the number of differential feature branches; and *F_s_* denotes the feature extracted by the standard convolution branch. The differential convolution branches include central, angular, horizontal, and vertical difference convolutions, which capture gradient information along different directions. The weights *α_i_* were initialized to 0.5 and jointly optimized with the network during training.

Differential convolution can accentuate variations in hatching egg contours, beak edges, periocular regions, drinker boundaries, and feeder areas by computing response differences between adjacent pixels or local regions. DE-Conv responds more sensitively than a single standard convolution to local structural changes in complex backgrounds. This sensitivity helps distinguish hatching eggs from litter, duck beaks from feathers, and drinking movements from stationary head postures.

Feature processing in C3K2 was initialized by mapping and splitting the input using convolution branches. Bottleneck structures incorporating DE-Conv extracted deep features, followed by cross-layer fusion via feature concatenation. The design retained the advantages of cross-stage partial connections in C3K2 while improving network responses to small targets and their edges. For partially occluded or low-contrast hatching eggs and duck beaks, DE-C3K2 strengthened locally salient features, yielding more discriminative inputs for subsequent multi-scale feature fusion and detection head prediction.

#### 2.2.2. Optimization of Multi-Scale Target Detection

Target scales vary considerably in breeding duck houses. Farther from the camera, hatching eggs and duck beaks often span only a few pixels, whereas nearby duck heads, bodies, and egg-laying postures span a much wider range of scales. Egg-laying behavior also involves changes in posture, head pitching, neck stretching, and partial occlusion. Consequently, targets within the same category can vary considerably in scale over time and across locations.

**Figure 3:**
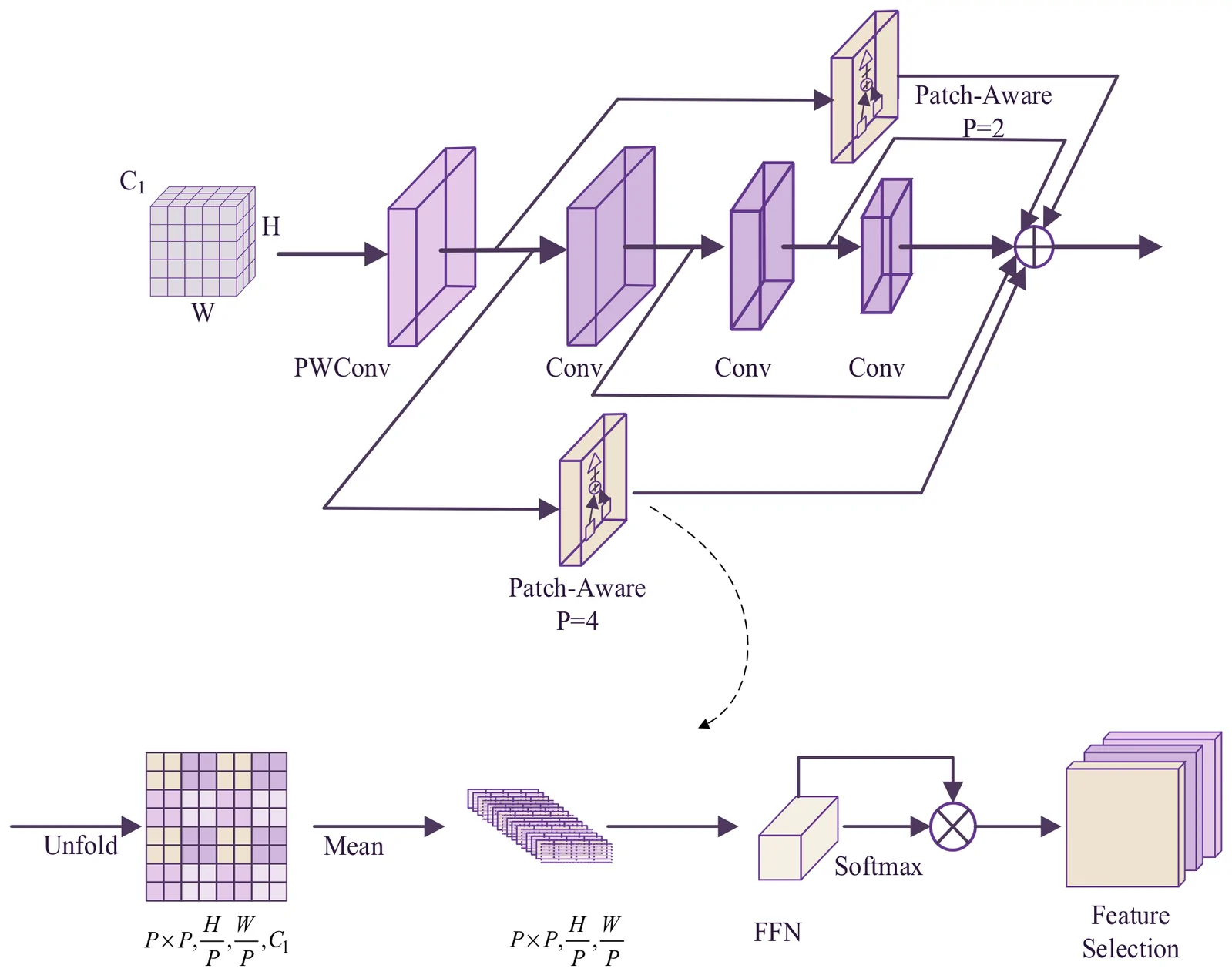
Structure of the Parallelized Patch-Aware Attention (PPA) module.

In YOLO26, SPPF primarily expands the receptive field and integrates contextual information across levels, but its ability to balance local details and global semantics dynamically remains somewhat limited. To improve the detection of duck behavior targets across scales, we inserted a PPA module [22] at the end of the backbone, before SPPF. Figure 3 shows its structure.

PPA uses parallel local perception, global context, and deep convolution feature branches to model information at different spatial scales. For an input feature map *F*, Equation (2) expresses the PPA operation.

(2) $$F_{\mathrm{ppa}}=A(F_{l}(F),F_{g}(F),F_{s}(F))$$

where $F_{l}$, $F_{g}$, and $F_{s}$ represent the local, global, and serial convolution feature extraction branches, respectively; A denotes the attention weighting and feature fusion operation; and *F_ppa_* denotes the enhanced PPA output. The input features are first adjusted by pointwise convolution. The local and global branches compute patch-aware attention over non-overlapping patches with sizes of *P* = 2 and *P* = 4, respectively. The serial branch consists of three stacked 3 × 3 convolutions. The branch outputs are summed and subsequently refined by channel and spatial attention.

The local feature branch captures textures, contours, and edges within small regions, improving recognition of hatching egg boundaries, beak tips, drinker edges, and local feeder areas. The global branch aggregates semantic context over a wider area. This helps the model represent spatial relationships among ducks, feeders, drinkers, and nest boxes, thereby reducing false detections caused by background textures. Through successive convolution layers, the serial branch extracts more abstract semantic features and improves the representation of duck postures and behavioral states.

Introducing PPA before SPPF helped fully retain and reinforce key information across scales before pooling over a large receptive field. PPA also improved the representation of tiny targets, such as distant hatching eggs and duck beaks, while preserving global semantic information for larger targets, including duck heads, bodies, and egg-laying postures. Hence, it could improve the robustness of feeding, drinking, and egg-laying behavior detection across scales in complex housing environments.

#### 2.2.3. Model Lightweighting

Deployment on breeding duck farms generally requires detection models to run on edge computing devices, embedded GPUs, or smart camera terminals. Therefore, improvements in detection accuracy should be balanced against parameter count, computational cost, and inference latency. The YOLO26 neck combines a feature pyramid network with a path aggregation network to fuse features bidirectionally across levels. However, redundant computation in some conventional convolution modules limits real-time operation on resource-constrained devices.

**Figure 4:**
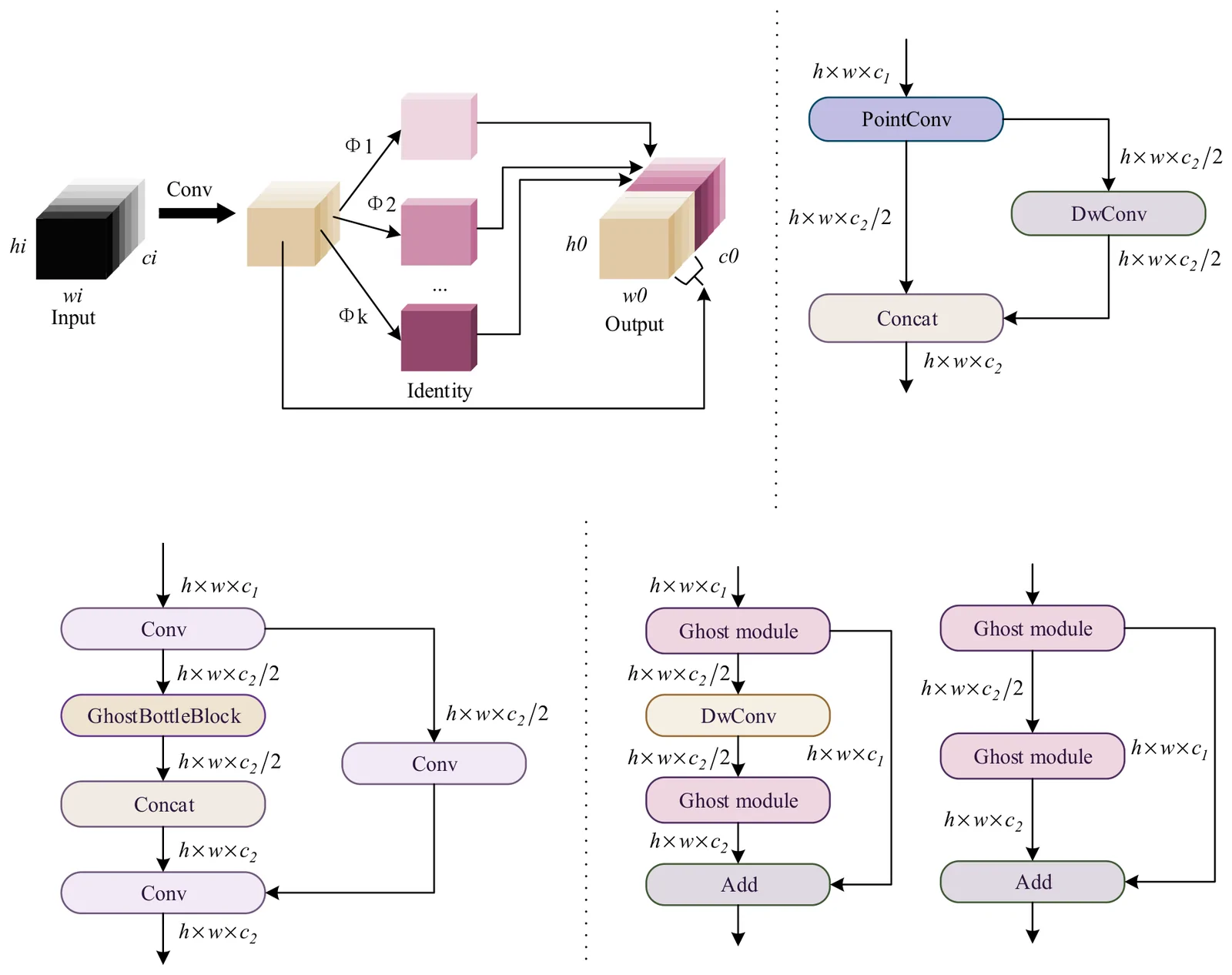
Structures of the GhostBottleneck and C3Ghost modules.

To reduce model complexity, C3Ghost structures replaced all four C3K2 feature fusion modules in the neck (Figure 2). Two were in the top-down path after upsampling and concatenation, and two were in the bottom-up path after downsampling and concatenation. All backbone modules were left unchanged during this substitution. C3Ghost used Ghost convolution [23] to generate primary features and then constructed redundant features through inexpensive linear transformations, substantially reducing repeated computation in conventional convolutions. Figure 4 shows the GhostBottleneck and C3Ghost structures.

GhostBottleneck consists of two stacked GhostModules. The first expands the feature channels, whereas the second compresses them to match the shortcut branches. When the stride was 2, the two GhostModules were connected by a stride-2 depthwise convolution (DWConv) for downsampling. The shortcut branch was then added element-wise to the GhostModule output to preserve shallow feature information. Batch normalization (BN) followed each GhostModule. Rectified linear unit (ReLU) activation followed every layer, except the second GhostModule, ensuring nonlinear feature representation and training stability. This GhostBottleneck follows the original GhostNet design. Our implementation differs only in its integration within the CSP-based C3 structure and in the substitution locations specified above.

C3Ghost retained the feature reuse provided by the CSP structure while replacing some standard bottleneck convolutions with GhostBottleneck structures, enabling multi-scale feature fusion at a lower computational cost. In duck behavior detection, the neck primarily combined shallow localization details with deep semantic information. C3Ghost reduced redundant computation while maintaining effective representations of hatching egg edges, local beak textures, and duck postures.

This modification was well suited for continuous on-farm monitoring. Reducing parameter count and floating-point operations improves inference efficiency while maintaining detection performance. The resulting lightweight model can support subsequent real-time behavior analysis, abnormal behavior alerts, and automated hatching egg counting in breeding ducks.

### 2.3. Quantification Methods for Egg Production Performance

To further evaluate the practical value of DPG-YOLO for monitoring egg-laying behavior and counting hatching eggs, we quantified egg production indicators from the video detection results. Combining object detections with temporal information enabled calculation of daily laying behavior frequency and daily egg production rate, among other indicators, to assess egg production performance automatically.

#### 2.3.1. Quantification of Egg-Laying Behavior Frequency

Frames were processed using DPG-YOLO. One nest-box occupancy event was defined as a breeding duck entering a nest box and remaining inside. Events separated by detection gaps no longer than a preset threshold were merged to prevent repeated counting caused by brief occlusions, missed detections, or detection interruptions. Let *t_in_*_,*j*_ and *t_out_*_,*j*_ denote the entry and departure times for the *j*-th occupancy event. The occupancy duration *D_j_* is defined in Equation (3). The valid duration range was set to [*T_min_*, *T_max_*]. The *j*-th occupancy event was classified as a valid egg-laying event when two conditions were met: *T_min_* ≤ *D_j_* ≤ *T_max_*, and a newly laid hatching egg was detected during occupancy or after the duck left the nest box. The daily total of identified occupancy events was denoted by *J*. For the *j*-th event, validity was encoded by *L_j_*, with *L_j_* = 1 for a valid egg-laying event and *L_j_* = 0 otherwise. The daily number of valid egg-laying events, *N_lay_*, is defined in Equation (4).

*D_j_* = *t_out_*_,*j*_ − *t_in_*_,*j*_ (3)

(4) $$N_{\mathrm{lay}}=\sum_{j=1}^{J}L_{j}$$

#### 2.3.2. Quantification of Egg Production Rate

Daily hatching egg counts were obtained from video images captured at 08:00. Frames were extracted within a preset 10 s window centered at 08:00 each day and processed by DPG-YOLO. Frames were considered valid when the nest-box area was clearly visible without severe motion blur, abnormal exposure, or transient occlusion, such as a duck crossing beneath the lens. Frames that failed these criteria were excluded before counting.

This time point was selected based on the laying rhythm of breeding ducks and the farm’s management schedule. Egg laying peaked at approximately 03:00–04:00 and was essentially complete by early morning, with no significant increase in egg numbers thereafter. Farm staff collected eggs shortly after 08:00 each day. Therefore, a snapshot was taken immediately before daily egg collection, ensuring that the visible eggs approximated the full daily output and that none had been removed before counting. After the model produced a bounding box and confidence score for each target, non-maximum suppression removed duplicate boxes for the same egg.

DPG-YOLO processes each frame independently, without temporal dependence between frames. A 5 s interval was used only during dataset construction to reduce redundancy among highly similar consecutive frames. During inference, all frames within the 10 s window of video recorded at 25 frames/s were processed. These repeated observations were used to determine the daily egg count from the mode. This yielded the hatching egg count *n_i_* for the *i*-th valid image. For *M* valid frames in the window, the mode of the detected counts was used as *N_egg_*, the final hatching egg count for that day, as defined in Equation (5). This procedure yielded seven daily records over the collection period. The 08:00 snapshots from 24 to 30 August summarized eggs laid during the preceding day and night, corresponding to laying days from 23 to 29 August. The daily egg production rate (*ER*) was calculated by dividing the number of hatching eggs detected that day by the number of female breeding ducks in the experimental area (Equation (6)).

*N_egg_* = *Mode*(*n*_1_,*n*_2_,…,*n_M_*) (5)

where Mode(·) returns the most frequently occurring egg count. In the rare case of a tie, the smallest tied value was used as a conservative estimate of daily egg count.

(6) $$\mathrm{ER}=\frac{N_{\mathrm{egg}}}{N_{\mathrm{duck}}}\times 100\%$$

where *N_duck_* represents the number of female breeding ducks in the experimental area.

### 2.4. Experimental Setup and Evaluation Metrics

The dataset was randomly partitioned into training, validation, and test sets at a ratio of 7:1:2 across the full 7-day collection period, with all recording days represented in each partition. Two measures reduced redundancy between partitions. First, frames were extracted at 5 s intervals, equivalent to 125 frames at 25 frames/s, during which duck postures and nest occupancy typically changed. Second, image cleaning discarded most temporally adjacent frames, leaving the retained images irregularly spaced in time. Mosaic augmentation was applied only to the training partition after splitting. The training hardware comprised an Intel i9-14900K CPU (Intel, Santa Clara, CA, USA), an NVIDIA RTX 4090 GPU (NVIDIA, Santa Clara, CA, USA), and 128 GB of memory. The model was trained for 200 epochs with a batch size of 16, using SGD with a momentum of 0.9 and an initial learning rate of 0.001. PyTorch 2.4.1 was used for training, and CUDA 12.6 supported parallel computing.

Detection performance was assessed using precision (P), recall (R), and mean average precision (mAP). Computational efficiency was quantified using floating-point operations (FLOPs), reported in billions (GFLOPs), model size (MB), and inference speed in frames per second (FPS). Model size scales linearly with the number of parameters and thus directly reflects parameter count. These metrics are shown in Equations (7)–(11).

(7) $$\mathrm{Precision}=\frac{\mathrm{TP}}{\mathrm{TP}+\mathrm{FP}}$$

(8) $$\mathrm{Recall}=\frac{\mathrm{TP}}{\mathrm{TP}+\mathrm{FN}}$$

(9) $$F1=2\times \frac{\mathrm{Precision}\times \mathrm{Recall}}{\mathrm{Precision}+\mathrm{Recall}}$$

(10) $$\mathrm{AP}=\int_{0}^{1}P(R)\mathrm{dR}$$

(11) $$\mathrm{mAP}=\frac{\sum_{i=1}^{N}AP_{i}}{N}$$

Specifically, true positives (TP) are positive samples correctly identified as positive. False negatives (FN) are positive samples incorrectly predicted as negative. False positives (FP) are negative samples incorrectly predicted as positive. True negatives (TN) are negative samples correctly identified as negative.

## 3. Results

### 3.1. Ablation Experiments

We conducted ablation experiments to evaluate the contribution of each modified module to DPG-YOLO performance in detecting duck behavior and hatching eggs. Starting from the original YOLO26 baseline, we successively added DE-C3K2, the PPA multi-scale feature enhancement module, and the lightweight C3Ghost neck structure. Table 1 presents the results.

The original YOLO26 yielded precision, recall, and mAP50 values of 92.2%, 91.5%, and 92.4%, respectively, for duck behavior and hatching egg detection (Table 1). Adding DE-C3K2 to Group 2 increased these values to 93.1%, 92.5%, and 94.8%, representing gains of 0.9, 1.0, and 2.4 percentage points over the baseline, respectively. These results indicate that DE-C3K2 enhanced the network representation of edges, textures, and local morphological variations. By strengthening local differences between hatching eggs and complex litter backgrounds, the module reduced the risk of missed detections.

Introducing PPA before SPPF in Group 3 increased precision, recall, and mAP50 by 1.9, 1.9, and 1.4 percentage points, respectively. These results indicate that the local, global, and serial convolution branches of PPA could extract information across scales. This enabled simultaneous attention to the spatial semantic features of targets of different sizes, including egg-laying ducks and hatching eggs, thereby improving detection in complex housing environments.

Replacing conventional neck feature fusion modules with C3Ghost in Group 4 yielded the highest precision, recall, and mAP50 values: 96.8%, 96.3%, and 97.4%, respectively. These represented increases of 1.8, 1.9, and 1.2 percentage points over Group 3. C3Ghost also reduced computational cost from 9.2 to 7.6 GFLOPs (17.4%) and increased inference speed from 112.0 to 124.1 frames/s (10.8%). Thus, C3Ghost reduced redundant computation in neck feature fusion, allowing greater attention to discriminative information such as hatching egg contours, duck postures, and laying nest regions. Detection performance improved as computational complexity decreased.

Compared with the YOLO26 baseline, DPG-YOLO increased precision, recall, and mAP50 by 4.6, 4.8, and 5.0 percentage points, respectively. Despite an increase in computational cost of 0.8 GFLOPs, the model achieved an inference speed of 124.1 frames/s. These results indicate that DE-C3K2 enhanced small-target features, PPA extracted multi-scale information, and C3Ghost provided lightweight feature fusion. The three modules complemented one another, balancing detection accuracy, computational complexity, and real-time inference speed in complex breeding duck housing environments.

### 3.2. Comparison with Other Object Detection Models

We compared DPG-YOLO with widely used object detection models to evaluate its suitability for monitoring egg-laying behavior and detecting hatching eggs in breeding ducks. Comparisons covered detection accuracy, computational efficiency, and visual detection results in representative scenarios.

#### 3.2.1. Comparison of Detection Accuracy and Computational Efficiency

**Figure 5:**
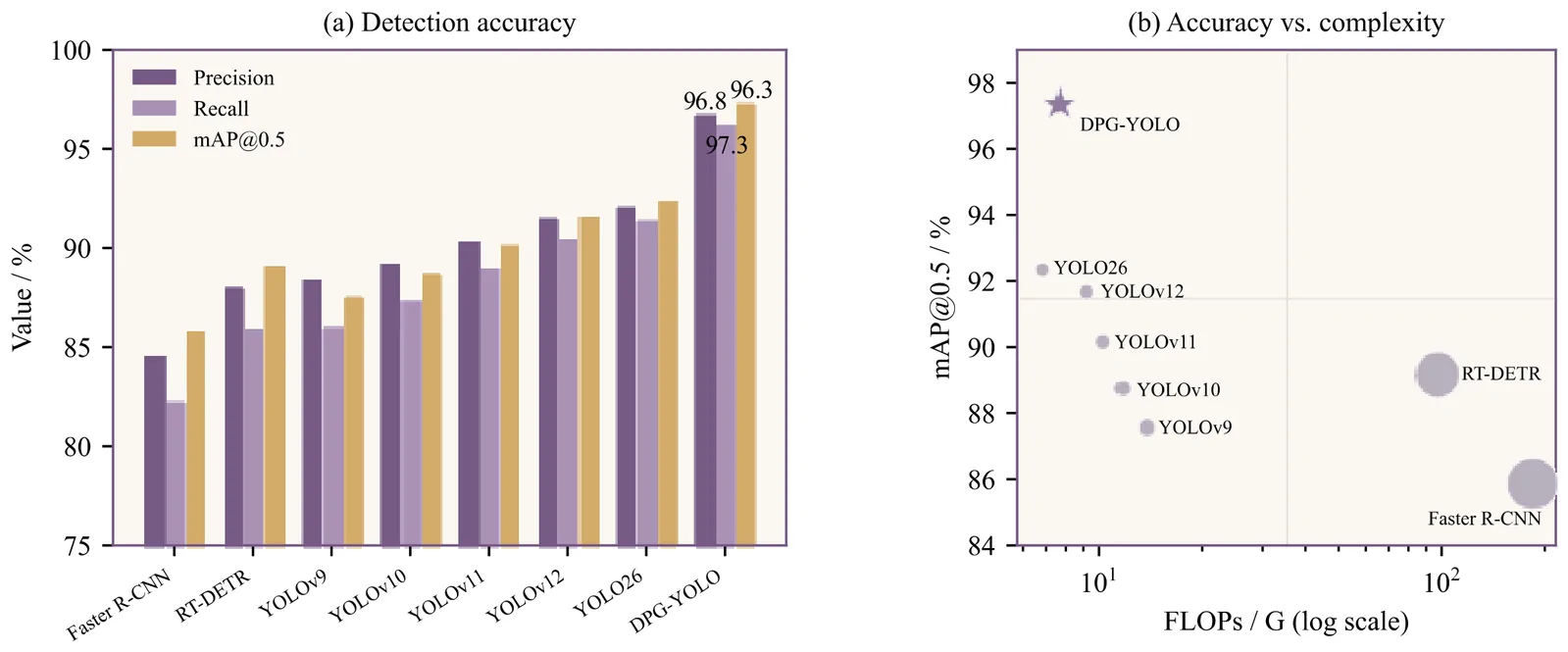
Comparison of detection accuracy and efficiency among different models: (**a**) detection accuracy; (**b**) computational efficiency. Note: in (**b**), the FLOPs axis is logarithmic, and the marker size is proportional to model size (MB).

Faster R-CNN, RT-DETR, YOLOv9, YOLOv10, YOLOv11, YOLOv12, YOLO26, and DPG-YOLO were compared for egg-laying behavior and hatching egg detection. Accuracy metrics included precision, recall, and mAP50, and efficiency metrics included parameter count, computational cost, model size, and inference speed. These metrics were used to assess each model’s detection performance, computational complexity, and capacity for real-time deployment. Figure 5 and Table 2 present the comparisons.

Faster R-CNN had the lowest overall detection performance, with precision, recall, and mAP50 values of 84.6%, 82.3%, and 85.9%, respectively (Figure 5a). RT-DETR outperformed Faster R-CNN, and the YOLO models achieved higher detection accuracy. YOLO26 achieved 92.2%, 91.5%, and 92.4% for the three metrics, surpassing YOLOv9, YOLOv10, YOLOv11, and YOLOv12. DPG-YOLO performed best, with precision of 96.8%, recall of 96.3%, and mAP50 of 97.4%. Its respective gains of 4.6, 4.8, and 5.0 percentage points over YOLO26 indicated reductions in false and missed detections.

Figure 5b and Table 2 compare computational complexity, model size, and detection efficiency. Faster R-CNN and RT-DETR required 182.6 and 96.3 GFLOPs and processed only 18.7 and 32.4 frames/s, respectively, making them less suitable for real-time edge deployment. YOLO26 required 6.8 GFLOPs, occupied 6.2 MB, and processed 135.4 frames/s. DPG-YOLO required slightly more computation and storage, at 7.6 GFLOPs and 7.1 MB, but still processed 124.1 frames/s, meeting the real-time demands of continuous video analysis.

DPG-YOLO achieved higher detection accuracy with only a slight increase in computational overhead. Collectively, DE-C3K2, PPA, and C3Ghost improved small-target representation and multi-scale feature modeling while reducing interference from complex backgrounds. This balance of accuracy, complexity, and inference speed can support reliable monitoring of egg-laying behavior and automated hatching egg detection in breeding ducks.

#### 3.2.2. Visual Comparison of Detection Results

**Figure 6:**
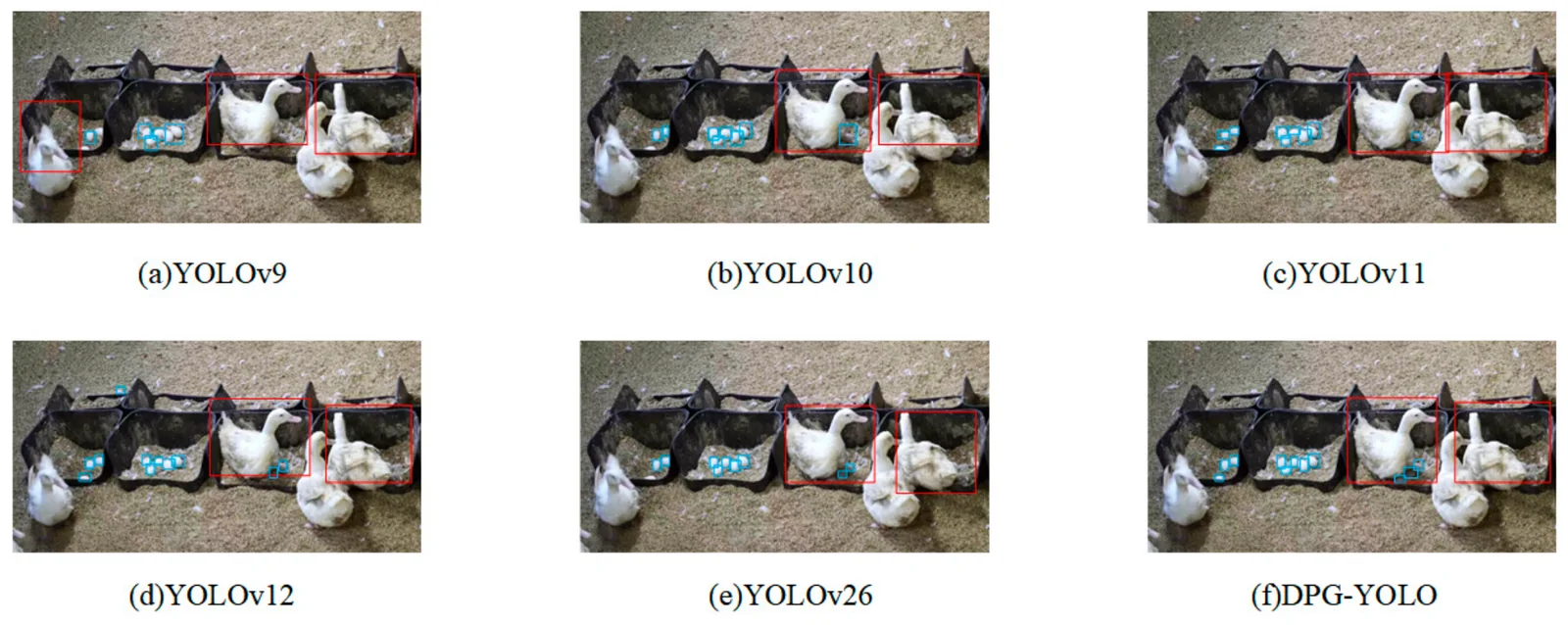
Visual comparison of detection results among different models.

Visual comparisons of YOLOv9, YOLOv10, YOLOv11, YOLOv12, YOLO26, and DPG-YOLO assessed hatching egg and egg-laying behavior detection. We examined detection completeness, localization accuracy, and false and missed detections. Figure 6 shows the results, with blue boxes marking hatching eggs and red boxes marking egg-laying behavior.

YOLOv9 detected some hatching eggs and instances of egg-laying behavior but missed several small eggs and targets in occluded areas (Figure 6a). It also produced false detections of egg-laying behavior. With an optimized detection head and end-to-end prediction mechanism, YOLOv10 localized targets more consistently than YOLOv9, and its bounding boxes covered the regions associated with egg-laying behavior more fully. However, it still missed hatching eggs near ducks that occluded one another (Figure 6b). The enhanced feature interaction and attention modeling enabled YOLOv12 to better detect densely clustered and partially occluded targets, thereby reducing the number of missed detections. However, a small number of missed and false detections of hatching eggs remained (Figure 6d).

Improved feature extraction and multi-scale information fusion allowed YOLOv11 to detect hatching eggs and egg-laying behavior more completely and localize bounding boxes more consistently. However, occasional missed detections and inaccurate boundary localization remained for complex backgrounds and where targets overlapped (Figure 6c). YOLO26, developed from YOLOv11, also detected hatching eggs and egg-laying behavior relatively completely. Some boxes only partially covered occluded targets, and a small number of detections were missed (Figure 6e). DPG-YOLO showed the best overall performance, detecting hatching eggs and egg-laying behavior more completely (Figure 6f). Its bounding boxes closely matched target boundaries, with almost no false or missed detections in these representative frames. Together with the full test set evaluation, these observations suggest greater robustness to complex litter backgrounds, occlusion of individual ducks, and variation in the target scale. However, occasional errors may occur under more challenging conditions. The model can support reliable monitoring of egg-laying behavior and accurate hatching egg counting on duck farms.

### 3.3. Statistical Analysis of Quantified Performance Indicators

To verify the reliability of DPG-YOLO estimates, we compared them with manually calculated values using independent test video segments as the units of analysis. Manual counts from the same videos at 08:00 served as the reference because they measured the same quantity as the automated estimates. This comparison isolated algorithmic error from management factors such as egg collection timing, broken eggs, and floor eggs. Spearman’s rank correlation analysis assessed whether values from the two methods varied consistently. The Wilcoxon signed-rank test assessed differences between paired values to examine systematic deviations in their absolute magnitudes. Together, these analyses evaluated the consistency of the automatically and manually calculated values. All analyses were performed using SPSS 26.0, with significance set at *p* < 0.05.

**Figure 7:**
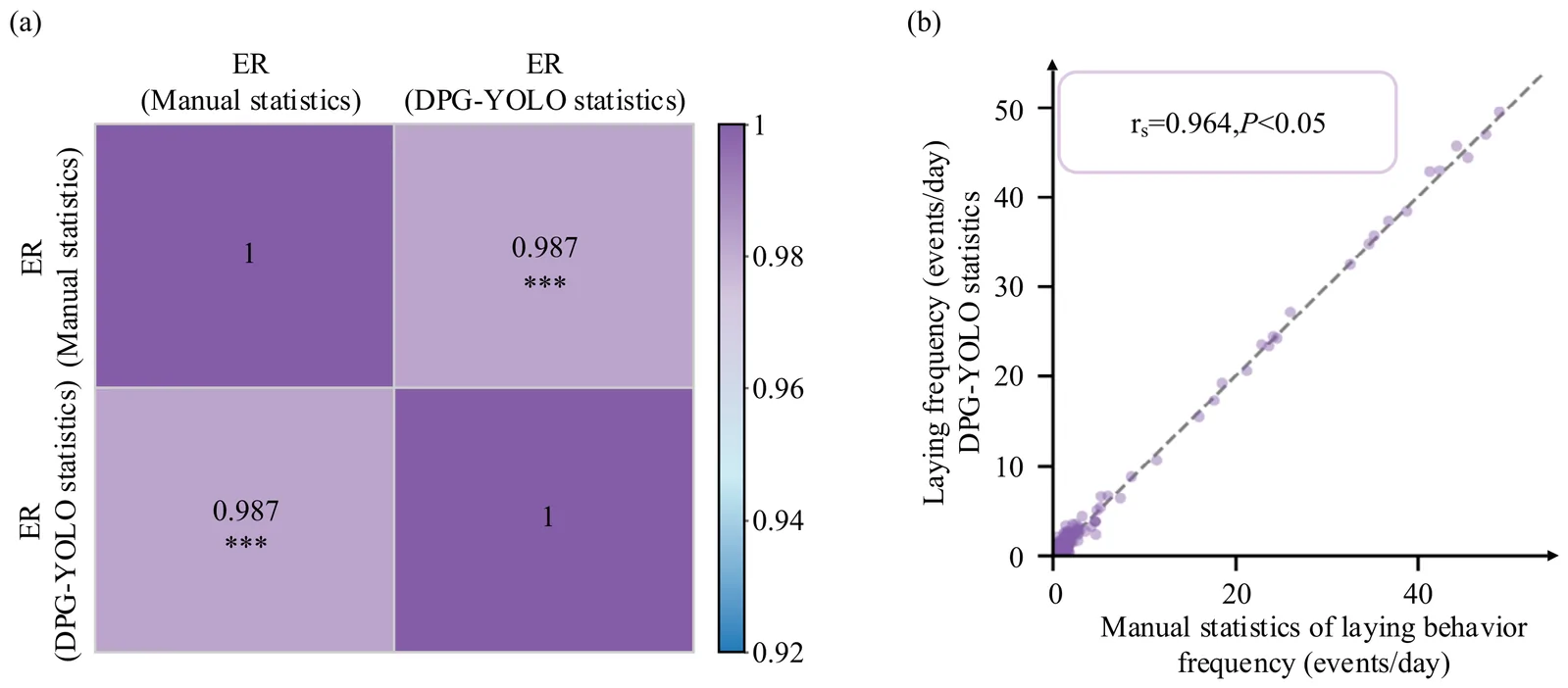
Spearman’s rank correlation analysis between DPG-YOLO and manual statistics: (**a**) egg production rate (*r_s_* = 0.987, *p* < 0.05); (**b**) laying behavior frequency (*r_s_* = 0.964, *p* < 0.05).

For egg production rate, Spearman’s rank correlation coefficient was 0.987 (*p* < 0.05), indicating an extremely strong positive correlation between the two methods (Figure 7). For laying behavior frequency, the coefficient was 0.964 (*p* < 0.05), indicating a significant positive correlation. These findings showed that the automatically and manually calculated values varied consistently. However, a high correlation alone does not establish the absence of bias in absolute values. Therefore, we also analyzed differences between the two methods.

**Figure 8:**
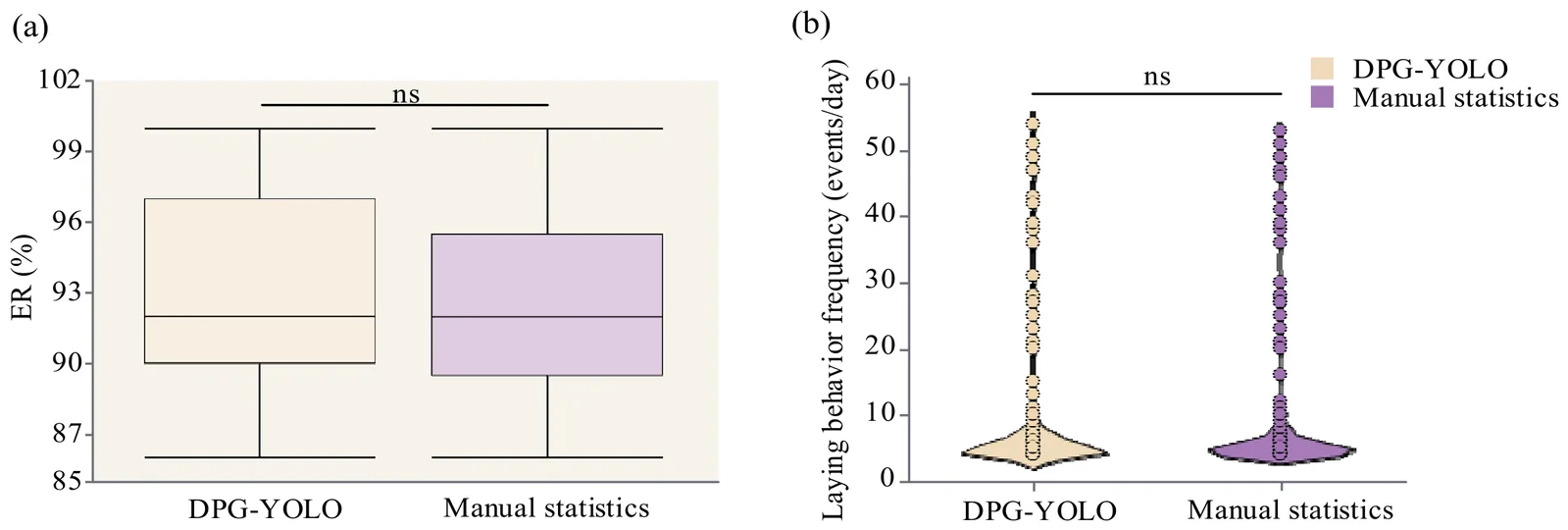
Difference analysis between DPG-YOLO and manual statistics (Wilcoxon signed-rank test): (**a**) egg production rate; (**b**) laying behavior frequency. ns indicates no significant difference (*p* > 0.05).

We examined agreement in absolute values by analyzing differences between automatically and manually calculated values. The Wilcoxon signed-rank test found no significant difference between methods for either egg production rate (*p* > 0.05; Figure 8) or laying behavior frequency (*p* > 0.05). Although values from the two methods could differ for individual samples, no statistically significant systematic bias was observed overall.

Collectively, the analyses showed good agreement in absolute values between DPG-YOLO estimates and manually calculated values. The model can reliably and automatically quantify egg production rate and egg-laying behavior frequency in breeding ducks. The estimated egg production rates (ER; approximately 87–99%) were consistent with the expected production level of 300-day-old breeding ducks at peak lay. These estimates can support continuous behavior monitoring, production performance evaluation, and management decisions in poultry production.

## 4. Discussion

By combining feature enhancement, multi-scale modeling, and lightweight fusion, DPG-YOLO established a workflow from detecting egg-laying behavior and hatching eggs to quantifying egg production performance automatically. Within DE-C3K2, differential convolution branches retained local features associated with abrupt grayscale changes, including hatching egg contours and duck beak edges. Adding this module increased mAP50 by 2.4 percentage points over the baseline, confirming the importance of fine-grained feature enhancement for detecting small targets against complex litter backgrounds. PPA integrated information across scales before pooling over a large receptive field, allowing distant hatching eggs and nearby duck bodies to be represented within one model despite their different scales. C3Ghost reduced redundant computation in the neck at an extremely low computational cost. Detection accuracy was maintained and improved while computational cost increased by only 0.8 GFLOPs, indicating that the lightweight reconstruction directed feature representation toward discriminative regions. Visual comparisons also showed that DPG-YOLO bounding boxes aligned more closely with hatching egg and egg-laying behavior target boundaries. Almost no false or missed detections occurred against complex litter backgrounds or under occlusion of individual ducks in the representative examples in Figure 6. However, these selected frames provide illustrative qualitative evidence. They visually support the effectiveness of the structural modifications and complement the quantitative evaluation on the full test set.

This study differs from previous research in three respects. First, DPG-YOLO simultaneously detected egg-laying behavior and hatching eggs from a single top-down view under commercial breeding conditions. Previous duck-focused studies [24,25] addressed behavior recognition and egg counting as separate tasks. Previous systems typically used separate setups for behavior recognition and egg detection. Second, we quantified egg production performance by identifying nest-box occupancy events and taking the mode of egg counts across frames. This converted frame-level detections into production indicators relevant to animal husbandry, including daily egg production rate and laying behavior frequency. Third, we evaluated automatically calculated values using Spearman’s rank correlation analysis and the Wilcoxon signed-rank test. For both indicators, automated estimates were significantly and positively correlated with manually calculated values, with no significant differences between methods. These results demonstrated high consistency in variation patterns and absolute values, supporting the use of model outputs in production records. The capacity to produce usable records is a prerequisite for the practical deployment of vision-based poultry monitoring technologies.

This study had several limitations. Data were collected from one farm and one breed over seven days using image-level partitioning. Therefore, generalization across housing environments and residual temporal correlation between partitions require evaluation in multi-farm studies using day-wise partitioning. Egg-laying events were identified by combining nest-box occupancy with newly laid egg detection, a rule that cannot fully exclude non-laying occupancy. The daily egg production rate was estimated from a single 08:00 snapshot and therefore approximated the daily output. Validation was limited to day-level agreement with manually calculated values over seven records (n = 7). Event-level precision and recall were not evaluated. Individual tracking and on-device deployment were not conducted. Future work will expand data collection across farms and seasons, use day-wise partitioning, and incorporate multi-object tracking with finer-grained event annotations. The estimates will also be calibrated against long-term farm records, and edge deployment tests will be conducted to support practical use in breeding duck production.

## 5. Conclusions

This study developed DPG-YOLO to detect egg-laying behavior and hatching eggs in breeding ducks under conditions involving litter interference, small eggs, variable duck postures, and occlusion. Reconstructing DE-C3K2, adding the PPA multi-scale feature enhancement module, and adopting C3Ghost for lightweight feature fusion improved the extraction of discriminative features. These features included hatching egg edges, laying nest regions, and duck postures associated with laying. Precision, recall, and mAP50 reached 96.8%, 96.3%, and 97.4%, exceeding the YOLO26 baseline by 4.6, 4.8, and 5.0 percentage points, respectively. With a computational cost of 7.6 GFLOPs and a model size of 7.1 MB, DPG-YOLO processed 124.1 frames/s, balancing detection accuracy and real-time operation. Automatically estimated egg production rates and laying behavior frequencies were significantly and positively correlated with manually calculated values (*r_s_* = 0.987 and 0.964, *p* < 0.05). Neither indicator differed significantly between methods (*p* > 0.05). DPG-YOLO can provide a reliable technical basis for automated collection of egg-laying information, evaluation of reproductive performance, and precision feeding management in breeding duck production.

## Figures and Tables

**Table 1:** Results of the ablation experiments for DPG-YOLO.

Group	YOLO26	DE-C3K2	PPA	C3Ghost	Precision/%	Recall/%	mAP50/%	FLOPs/G	FPS/(Frames/s)
1	√	-	-	-	92.2	91.5	92.4	6.8	135.4
2	√	√	-	-	93.1	92.5	94.8	7.3	125.0
3	√	√	√	-	95.0	94.4	96.2	9.2	112.0
4	√	√	√	√	96.8	96.3	97.4	7.6	124.1

**Table 2:** Computational efficiency comparison of different detection models.

Model	FLOPs/G	Model Size/MB	FPS/(Frames/s)
Faster R-CNN	182.6	158.4	18.7
RT-DETR	96.3	126.5	32.4
YOLOv9	13.7	10.8	97.2
YOLOv10	11.6	9.4	104.5
YOLOv11	10.2	8.6	112.8
YOLOv12	9.1	7.8	118.6
YOLO26	6.8	6.2	135.4
DPG-YOLO	7.6	7.1	124.1

## Data Availability

The dataset and code supporting the findings of this study are available from the corresponding author upon reasonable request, as public deposition is restricted owing to the commercial nature of the farm’s production data.

## Abbreviations

The following abbreviations are used in this manuscript:

CNN	Convolutional Neural Network
DE-C3K2	Detail-Enhanced C3K2
DPG-YOLO	The proposed lightweight detection model for breeding ducks
DWConv	Depthwise Convolution
ER	Egg Production Rate
FPS	Frames Per Second
GFLOPs	Giga Floating-Point Operations
mAP	mean Average Precision
P	Precision
PPA	Parallelized Patch-Aware Attention
R	Recall
R-CNN	Region-based Convolutional Neural Network
RT-DETR	Real-Time Detection Transformer
rs	Spearman’s Rank Correlation Coefficient
SPPF	Spatial Pyramid Pooling—Fast
YOLO	You Only Look Once
